# High RAS-related protein Rab-7a (RAB7A) expression is a poor prognostic factor in pancreatic adenocarcinoma

**DOI:** 10.1038/s41598-022-22355-1

**Published:** 2022-10-19

**Authors:** Qi Liu, Yang Bai, Xiaoyi Shi, Danfeng Guo, Yong Wang, Yun Wang, Wen-zhi Guo, Shuijun Zhang

**Affiliations:** 1 Department of Hepatobiliary and Pancreatic Surgery, The First Affiliated Hospital of Zhengzhou University, No.1 Jianshe Road, Zhengzhou, 450052 Henan China; 2 Henan Diagnosis & Treatment League for Hepatopathy, Zhengzhou, 450052 China; 3 Henan Engineering & Research Center for Diagnosis and Treatment of Hepatobiliary and Pancreatic Surgical Diseases, Zhengzhou, 450052 China; 4 Zhengzhou Key Laboratory for Hepatobiliary & Pancreatic Diseases and Organ Transplantation, Zhengzhou, 450052 China; 5 Department of Anesthesiology, Pain and Perioperative Medicine, The First Affiliated Hospital of Zhengzhou University, Zhengzhou, 450052 China

**Keywords:** Cancer, Medical research

## Abstract

Pancreatic adenocarcinoma (PAAD) is a frequent type of cancer in adults worldwide, and the search for better biomarkers is one of the current challenges. Although RAB7A is associated with tumour progression in multiple tumour types, there are only a few reports in PAAD. Therefore, in this paper, RNA sequencing data were obtained from TCGA(The Cancer Genome Atlas) and GTEx to analyse RAB7A expression and differentially expressed genes (DEGs) in PAAD. The functional enrichment of RAB7A-associated DEGs was analysed by protein‒protein interaction (PPI) networks, immune cell infiltration analysis and GO/KEGG analyses. Additionally, Kaplan‒Meier and Cox regression analyses were used to determine the clinical significance of RAB7A in PAAD. High RAB7A expression was associated with poor prognosis in 182 PAAD specimens, including subgroups of patients aged ≤ 65 years, with male sex, not receiving radiotherapy, and with a history of previous alcohol consumption (P < 0.05). Cox regression analysis showed that elevated RAB7A was an independent prognostic factor, and the prognostic nomogram model included radiotherapy status, presence of postoperative tumour residual and histologic grade. Overall, RAB7A overexpression may serve as a biomarker for poor outcome in pancreatic cancer. The DEGs and pathways revealed in this work provide a tentative molecular mechanism for the pathogenesis and progression of PAAD.

## Introduction

Pancreatic cancer is a highly malignant tumour of the digestive system. According to the International Agency for Research on Cancer (IARC), pancreatic cancer is the 14th most common malignancy in the world, causing approximately 430,000 deaths each year, and is the seventh leading cause of cancer-related deaths^1,2^. The latest statistics for 2021 show that pancreatic cancer is the 10th most common new malignancy in men, the 9th most common in women, and the 4th most common cause of cancer-related death in the United States^2^. In 2017, the National Cancer Centre of China reported that pancreatic cancer was the 7th and 11th leading cause of malignant tumour incidence among men and women in China, respectively, and the 6th leading cause of malignancy-related deaths^3^. The incidence and mortality rates of pancreatic cancer have become a serious threat to human life and health. In recent years, the 5-year survival rate for pancreatic cancer has increased from 5 to 10% thanks to the use of comprehensive oncology treatments, but the improvement in the survival rate for pancreatic cancer patients has not been significant^4^. Currently, radical surgical resection is still the main treatment strategy for pancreatic cancer. However, the insidious clinical presentation of pancreatic cancer and the lack of early diagnostic strategies mean that most patients are diagnosed at an advanced stage, and more than 80% of patients are unresectable and show extensive resistance to radiotherapy and insensitivity to immunotherapy, resulting in poor outcomes and a very poor prognosis^5^. Therefore, the identification of new biomarkers may contribute to a better understanding of the molecular basis of PAAD, which may play important roles in the diagnosis of PAAD, the prognostic stratification of patients, the prediction of the therapeutic response and the development of potential targeted drugs.

The RAS-related in brain (RAB) protein family consists of small guanosine triphosphatases (GTPases) that are mainly involved in membrane transport processes^6^. Rab7 is a small G protein in the Rab family. There are two types of Rab7 in mammals, Rab7a and Rab7b, which are 50% homologous and act at different stages of membrane transport^7^. Rab7a is mainly located in the late endosome and regulates the translocation of early endosomes to late endosomes and of late endosomes to lysosomes. Rab7a is involved in key aspects of mitochondrial and lysosomal interregulation, including regulating mitochondrial autophagosome formation, facilitating the fusion of mitochondrial autophagosomes with lysosomes and promoting mitochondrial-lysosome contact and dissociation. In addition to these functions, RAB7A has many other cellular functions, including autophagy^8^, apoptosis^6^, phagocytosis^9^, mitophagy and lipophagy^10^. Additionally, there are many reports in the literature suggesting that RAB7A is a major player in cancer^10^. However, to date, the expression of RAB7A in PAAD and its prognostic value remain unclear.

Thus, the purpose of this study was to establish a correlation between RAB7A expression levels and PAAD prognosis using the following steps. First, we analysed the expression of the core gene RAB7A using the RNA sequencing (RNA-seq) data of PAAD samples from The Cancer Genome Atlas (TCGA) and Genotype-Tissue Expression (GTEx) databases. Next, we examined the functional enrichment of RAB7A using protein‒protein interaction (PPI) networks, immune cell infiltration analyses, and GO/KEGG analyses. This allowed screening for significantly altered genes and pathways whose linkage to RAB7A may play a key role in PAAD development. Finally, we examined the clinical relevance of RAB7A in PAAD using Kaplan‒Meier analyses and Cox regression models, as well as the prognostic nomogram model.

## Materials and methods

### Human PAAD tissue specimens

Twelve pairs of fresh pancreatic cancer tissues and corresponding paracancerous tissues from patients who underwent pathological and clinical diagnosis in our department between July 2020 and August 2022 were collected for this study. All patients underwent surgery at the First Affiliated Hospital of Zhengzhou University, China. Ethics approval for the study protocol was obtained from the First Affiliated Hospital of Zhengzhou University.

### RNA sequencing data and bioinformatics analysis

RNA-seq data in TPM format for pan-cancer and PAAD samples from the TCGA and GTEx databases processed uniformly by the Toil process were obtained by UCSC XENA (https://xenabrowser.net/datapages/)^11^. The pan-cancer and PAAD data were extracted from TCGA (https://portal.gdc.cancer.gov/repository), and the corresponding normal tissue data were extracted from GTEx. This study complies completely with the established requirements of the TCGA and GTEx databases.

### RNA extraction and quantitative RT‑PCR

The total RNA of PAAD clinical specimens was extracted by TRIzol (Invitrogen, United States) and then reverse transcribed to cDNA via the HiScript® III First Strand cDNA Synthesis Kit (Vazyme, China). Then, 2 × SYBR Green qPCR Master Mix (Vazyme, China) was used for the qRT‒PCR assay. Relative mRNA expression levels were calculated with the comparative cycle threshold (CT) (2^−ΔΔCT^) method using β-actin as the endogenous control for normalization. The sequences of the primers for qRT‒PCR were as follows: β-actin forward: 5′- ACCTTCTACAATGAGCTGCG-3′ and reverse: 5′- CCTGGATAGCAACGTACATGG-3′; RAB7A forward: 5′-CCTCGAAAACAGACAAGTGGC-3′ and reverse: 5′- ATTCCGTGCAATCGTCTGGA-3′.

### Western blot (WB) analysis

Tissues were lysed with RIPA buffer supplemented with 1 mM PMSF (Solarbio, China) and placed on ice for 0.5 h. The protein concentrations were identified via a BCA protein analysis kit (Solarbio, China). The extracted proteins were separated by 10% SDS‒PAGE and transferred onto PVDF membranes (Millipore, Billerica, MA, USA). The following primary antibodies were used: RAB7A (Proteintech, Wuhan, China) and β-actin (Proteintech, Wuhan, China) antibodies. The following secondary antibodies were used: HRP-conjugated anti-rabbit IgG and anti-mouse IgG antibodies (Cell Signaling Technology, Danvers, MA, USA).

### Human protein atlas (HPA)

HPA contains immunohistochemical staining images of PAAD and normal tissues (https://www.proteinatlas.org/). HPA uses transcriptomics and proteomics to produce various protein profiles, such as cellular profiles, pathological profiles and tissue profiles. The HPAanalyse package in R was used to analyse the expression of RAB7A in all normal pancreatic and pancreatic cancer tissues on the HPA.

### Analyses of differentially expressed genes (DEGs)

To detect DEGs, we used the DESeq2 R package to compare the expression data (HTSeq-Count) of RAB7A at low and high levels of expression (cut-off value of 50%) in PAAD samples^12^. The top 5 DEGs with high and low expression levels were analysed using the heatmap method.

### Functional enrichment analysis

Functional enrichment analysis was performed for the DEGs with the thresholds of |logFC|> 1.5 and p.adj < 0.05. The ClusterProfiler package in R was used to perform functional analysis with Gene Ontology (GO) terms, including cellular component (CC), molecular function (MF), and biological process (BP) terms, and KEGG (Kyoto Encyclopedia of Genes and Genomes) pathway analysis^13–15^. The KEGG signalling pathway was analysed from www.kegg.jp/kegg/kegg1.html.

The GSEA method was used to determine functional and pathway differences between the high and low RAB7A expression groups with the ClusterProfiler package in R (3.14.3)^16^. Each analysis was performed with 5000 permutations of the gene set. Statistical significance was defined as adjusted P value < 0.05.

### Analyses of immune infiltration using single-sample gene set enrichment analysis (ssGSEA)

RAB7A immune infiltration analysis was performed in R (3.6.3) using the GSA package for ssGSEA, which included 24 immune cells: B cells; T cells; Tregs; Th2 cells; Th1 cells; Th17 cells; Tgd (T gamma delta) cells; Tfh (T follicular helper) cells; Tem (T effector memory) cells; Tcm (T central memory) cells; T helper cells; DCs; aDCs (activated DCs); iDCs (immature DCs); pDCs (plasmacytoid DCs); cytotoxic cells; eosinophils; CD8 T cells; macrophages; mast cells; NK cells; NK CD56bright cells; NK CD56dim cells; and neutrophils^17,18^. An analysis of the correlation between RAB7A and 24 immune cell enrichment scores was performed using Spearman correction. The enrichment scores of the high and low RAB7A expression groups were analysed using the Wilcoxon rank sum test.

### PPI network

The Search Tool for the Retrieval of Interacting Genes (STRING) database was used to construct the PPI network of DEGs^19^. The interaction score threshold was set at 0.4 as the cut-off criterion. Cytoscape (3.9.0) was used to map the PPI network, and MCODE (2.0.0) was used to identify the most important modules in the PPI network^20,21^. The selection criteria were MCODE score > 5, extent cut-off = 2, node score cut-off = 0.2, max depth = 100, and k-score = 2. Pathway and process enrichment analyses were performed using Metascape (https://metascape.org/gp/index.htm).

### Statistical analysis

All statistical analyses and graphs were performed using the ggplot2 package for R (3.3.3). To determine the level of RAB7A expression in unmatched specimens, the Wilcoxon rank sum test was used. To assess the connection between clinical features and RAB7A expression, the Kruskal‒Wallis test and Wilcoxon signed rank sum test were used. Cox regression analysis and the Kaplan‒Meier method were used to determine prognostic factors for patients. The effect of RAB7A expression on survival and other clinical features was assessed using multifactorial Cox analysis. In addition, ROC analysis was used to assess the effectiveness of high or low RAB7A expression in distinguishing healthy samples from PAAD samples, which was achieved using the pROC package in R (1.17.0.1). The calculated area under the curve (AUC) values ranged between 0.5 and 1.0, corresponding to a discriminating power of 50–100%.

### Clinical model construction and prediction

To personalize the prediction of overall survival (OS), progression-free interval (PFI) and disease-specific survival (DSS) for PAAD patients, the rms package (6.2–0) and survival package (3.2–10) of R were used to generate a nomogram containing salient clinical features and correction plots^22^. The concordance index (C-index) was used to calculate the prediction accuracy of the nomogram. The calibration curve can be graphically represented by the ratio of the probability predicted by the nomogram to the observed ratio, with a 45° diagonal being the most correct predicted value. All statistical tests were two-tailed, with 0.05 as the level of statistical significance.

### Ethics approval and consent to participate

This study had gained the informed consent from each subject and been approved by the Ethics Committee of the First Affiliated Hospital of Zhengzhou University. The ethical review number is 2019-KY-279.The ethical review certificate has been submitted in the related files section. The authors confirms that all experiments and public data acquisition were performed in accordance with relevant named guidelines and regulations.

## Results

### Expression of RAB7A across cancers and PAAD

**Figure 1:**
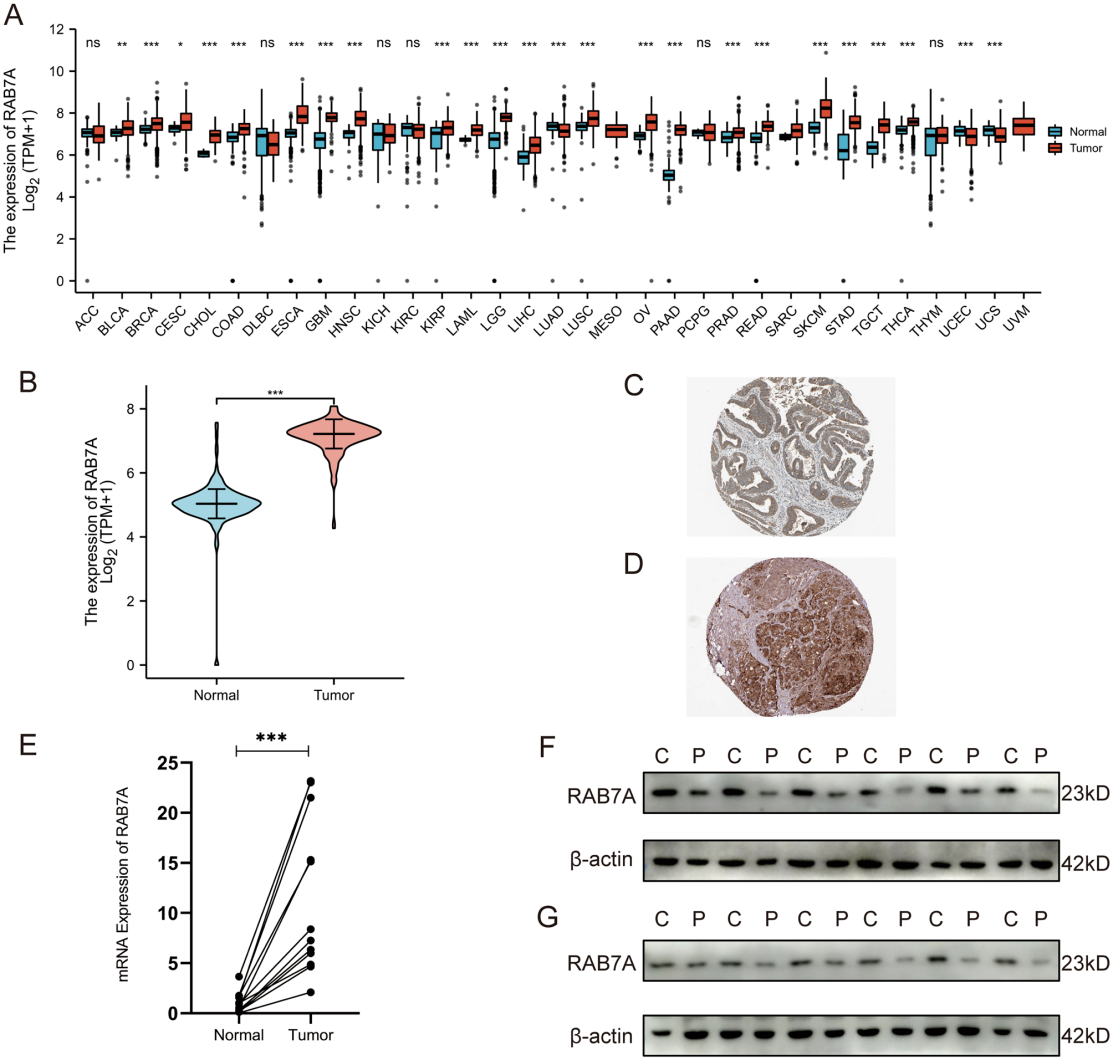
Expression of RAB7A in pan-cancer and PAAD. (**A**) The expression levels of RAB7A in various cancer tissues and their normal tissues. (**B**) Expression levels of RAB7A in paired PAAD tissues and normal pancreatic tissues. Comparative analysis of the two groups: Wilcoxon rank sum test; NS: P 0.05 or above; ***P < 0.001; **P < 0.01; *P < 0.05. (**C**) Protein expression levels of RAB7A in normal pancreatic tissue. (**D**) Protein expression level of RAB7A in PAAD specimens. (**E**) The mRNA expression level of RAB7A in 12 pairs of PAAD tissues and paraneoplastic tissues. (**F–G**) Western blot results of RAB7A in 12 pairs of PAAD tissues and paraneoplastic tissues. *C* cancer tissue, *P* paracancerous tissue.

UCSC XENA (https://xenabrowser.net/datapages/) RNA-seq data in TPM format from TCGA and GTEx were processed in unison by the Toil process (Vivian J et al.^11^). By comparing RAB7A expression in normal specimens in the TCGA and GTEx databases with tumour specimens in the corresponding TCGA database, RAB7A was found to be significantly highly expressed in 28 cancers (Fig. 1A), including pancreatic adenocarcinoma (PAAD) (Fig. 1B). In addition, RAB7A protein expression was analysed using the HPA database. We found that most types of cancers had positive staining for RAB7A. Images of normal pancreas (Patient ID: 1156) and pancreatic cancer (Patient ID: 4079) samples are provided here. The expression of RAB7A in normal pancreatic tissue was low to moderate, while it was highly expressed in pancreatic cancer (Fig. 1C,D). We analysed the expression of RAB7A in all normal pancreatic tissues versus all pancreatic cancer tissues on the human protein atlas. The results showed that RAB7A was moderately expressed in all normal pancreatic tissues and highly expressed in close to 75% of pancreatic cancer tissues (Supplementary Fig. 1). The same trend was found in our cohort of 12 pairs of PAAD tissues and paracancerous tissues. RAB7A transcript levels were shown to be relatively high in cancer tissues from PAAD patients, according to further investigation (Fig. 1E). Furthermore, RAB7A protein was also significantly upregulated in PAAD samples by western blot assay (Fig. 1F,G). Thus, these results suggest that RAB7A expression is significantly elevated in PAAD tissues.

### Identification of DEGs in PAAD samples using low and high RAB7A expression levels

**Figure 2:**
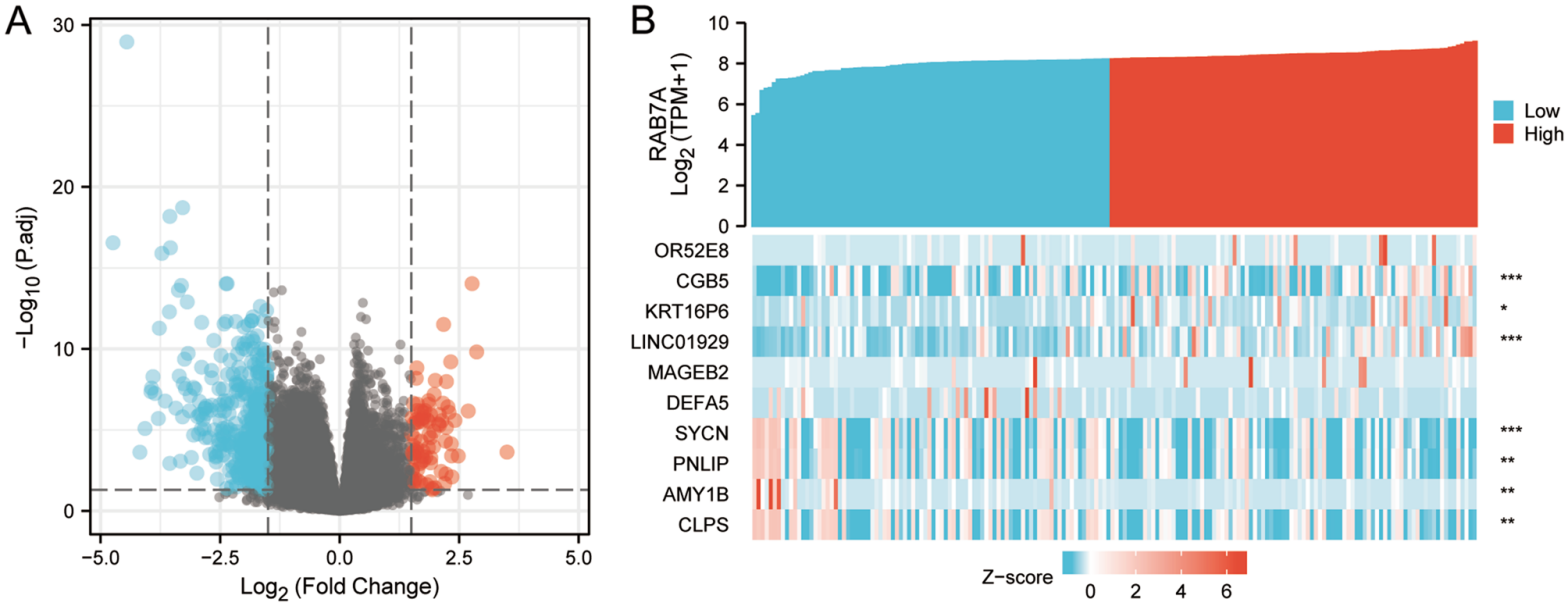
Between the groups with high RAB7A expression and those with low RAB7A expression, a total of 530 DEGs were confirmed to be statistically significant. (**A**) There are 84 up-regulated genes and 446 down-regulated genes in the volcano mapping of differentially expressed genes. The normalized expression levels are sorted from green to red in descending order. (**B**) A heat map of 10 differentially expressed RNAs, 5 of which are up-regulated and 5 of which are down-regulated. Green represents down-regulated genes; red represents up-regulated genes. The y-axis indicates differentially expressed RNA; the x-axis indicates the sample.

To determine differences in the median mRNA expression between the high and low expression groups, we analysed the gene expression profiles of each group. The current thresholds were |log fold change (logFC)|> 1.5 and p.adj < 0.05. The number of individuals meeting this threshold was 530, of which 84 had high RAB7A expression (positive logFC) and 446 had low RAB7A expression (negative logFC) (Fig. 2A). The heatmap illustrates the top five differentially expressed genes (DEGs) that were upregulated and the top five differentially expressed genes that were downregulated between the RAB7A high and low expression groups (Fig. 2B). The upregulated genes are: OR52E8, CGB5, KRT16P6, LINC01929, MAGEB2; the downregulated genes are: DEFA5, SYCN, PNLIP, AMY1B, CLPS.

### Functional enrichment analysis of DEGs

To obtain a greater understanding of the functional significance of the 530 DEGs between the high and low RAB7A expression groups in PAAD, we used the org.Hs.eg.db package in R to convert the IDs and the clusterProfiler tool in R to perform GO and KEGG functional enrichment analyses.

**Figure 3:**
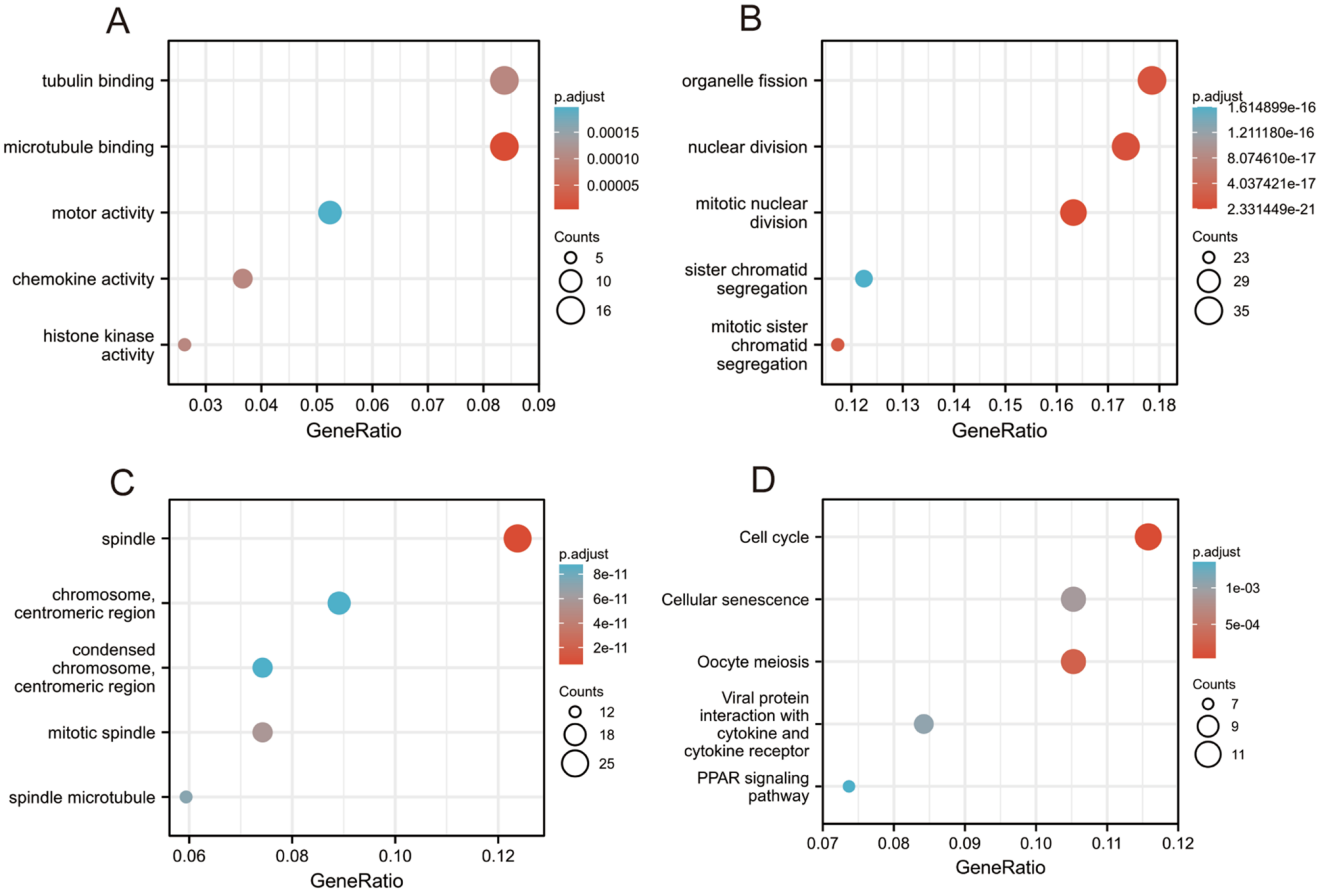
GO/KEGG enrichment analysis was used to determine the difference between high and low levels of RAB7A expression in TCGA patients. (**A**) GO terms enriched in the ‘Molecular Function’ category; (**B**) GO terms enriched in the ‘Biological Processes’ category; (**C**) Enriched GO terms in the “cellular component” category; (**D**) Annotations of KEGG pathways (Sourced from www.kegg.jp/kegg/kegg1.html). The X-axis indicates the percentage of DEGs, while the Y-axis indicates the various categories. Different colors denote various qualities, while various sizes denote the amount of DEGs.

The biological processes (BPs) were associated with the following: sister chromatid segregation, mitotic sister chromatid segregation, organelle fission, nuclear division and mitotic nuclear division; the cellular components (CCs) were associated with the following: centromeric region chromosome, spindle, condensed chromosome, mitotic spindle, spindle microtubule, and centromeric region; and the molecular functions (MFs) were associated with the following: microtubule binding, histone kinase activity, chemokine activity, motor activity, and tubulin binding. KEGG pathways included the cell cycle, viral protein interactions with cytokines and cytokine receptors, oocyte meiosis, cellular senescence and the PPAR signalling pathway (Fig. 3).

**Figure 4:**
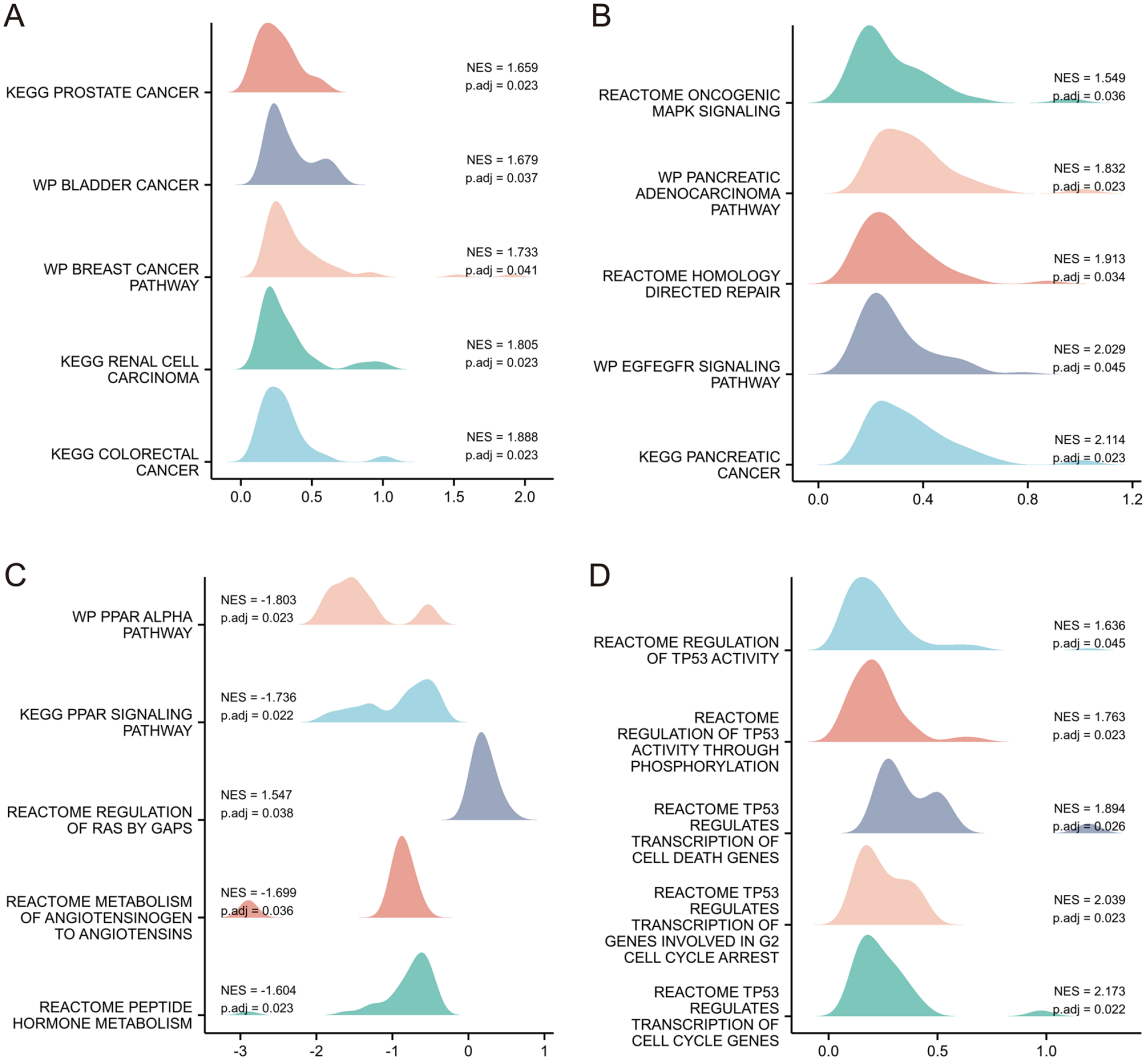
Plots of enrichment derived from the analysis of gene set enrichment (GSEA). (**A–D**) *p. adj* adjusted P-value, *ES* enrichment score, *NES* normalized ES.

With varying levels of RAB7A expression, GSEA was performed to delve deeper into the biochemical pathways implicated in PAAD. GSEA was used to determine the major signalling pathways implicated in PAAD by comparing the low and high RAB7A expression datasets. There were significant differences (p.adj < 0.05) in the enrichment of the MSigDB (C2.all.v7.0.symbols.gmt) collection for these pathways (Fig. 4).

### Immune infiltration analysis in PAAD

**Figure 5:**
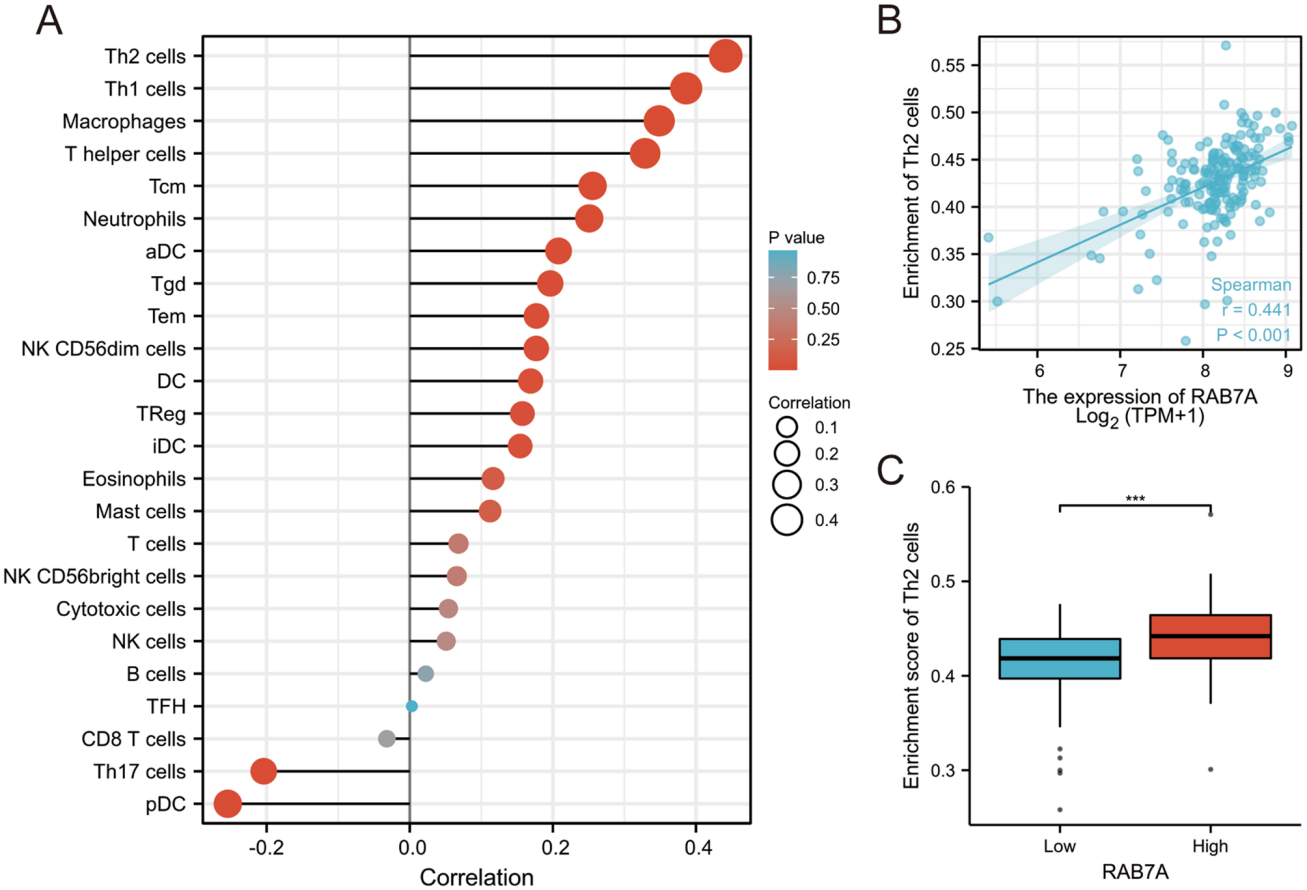
RAB7A expression levels were found to correlate with immune infiltration in the PAAD microenvironment. (**A**) RAB7A had a negative correlation with 3 immune cell subsets and a positive correlation with 21 immune cell subsets. (**B**) The relative enrichment of Th2 cells was positively correlated with the level of RAB7A expression (TPM). (**C**) Infiltration of Th2 cells between low and high RAB7A expression.

Spearman correlation analysis revealed a positive association between the level of RAB7A expression in the PAAD microenvironment and the level of immune cell infiltration as assessed by ssGSEA. RAB7A and 21 immune cell subpopulations had a positive association in forest plots, while RAB7A and 3 immune cell subpopulations had a negative correlation. Of these, the highest positive correlation was found between RAB7A and Th2 cells (Fig. 5). Therefore, we further analysed the relationship between the relative enrichment fraction of Th2 cells and the level of RAB7A expression (TPM) and the correlation between low and high RAB7A-expressing Th2 cell infiltration.

### Analyses of PPI enrichment in PAAD

**Figure 6:**
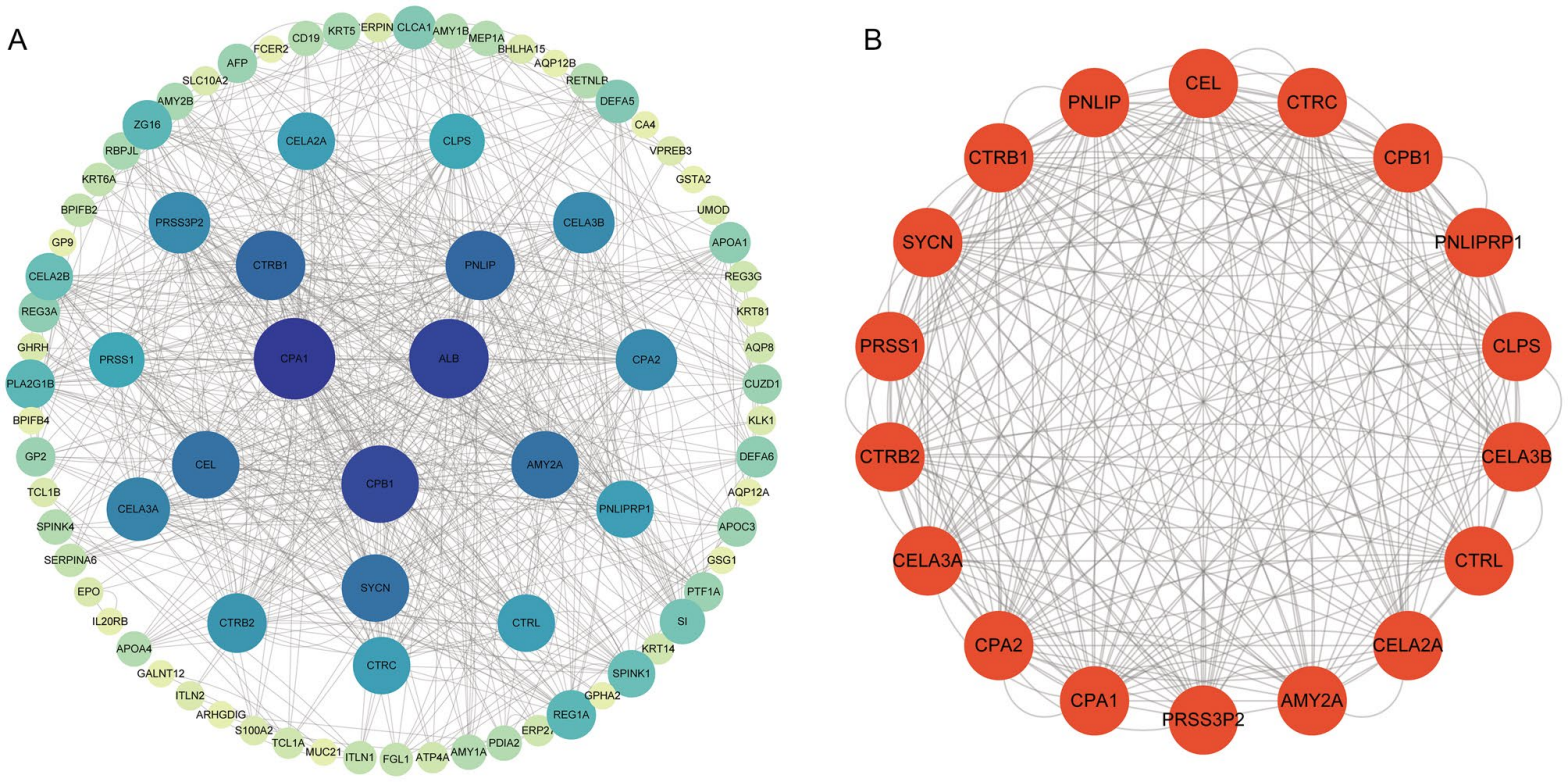
The PPI network of DEGs associated with RAB7A and the key modules contained inside. (**A**) The Cytoscape was used to design the DEG PPI network. (**B**) The most important module is the PPI network with 18 nodes and 260 edges.

STRING was used to establish a network of RAB7A and its putative coexpressed genes of the RAB7A-associated DEGs, and a total of 530 DEGs were screened (|log fold change (logFC)|> 1.5, p.adj < 0.05). MCODE in Cytoscape was used to visualize the PPI network with 82 nodes and 650 edges (Fig. 6A). The highest-scoring module had a score of 15.294 in MCODE and contained 18 nodes and 260 edges (Fig. 6B).

### Relationship between RAB7A expression and clinical features

**Figure 7:**
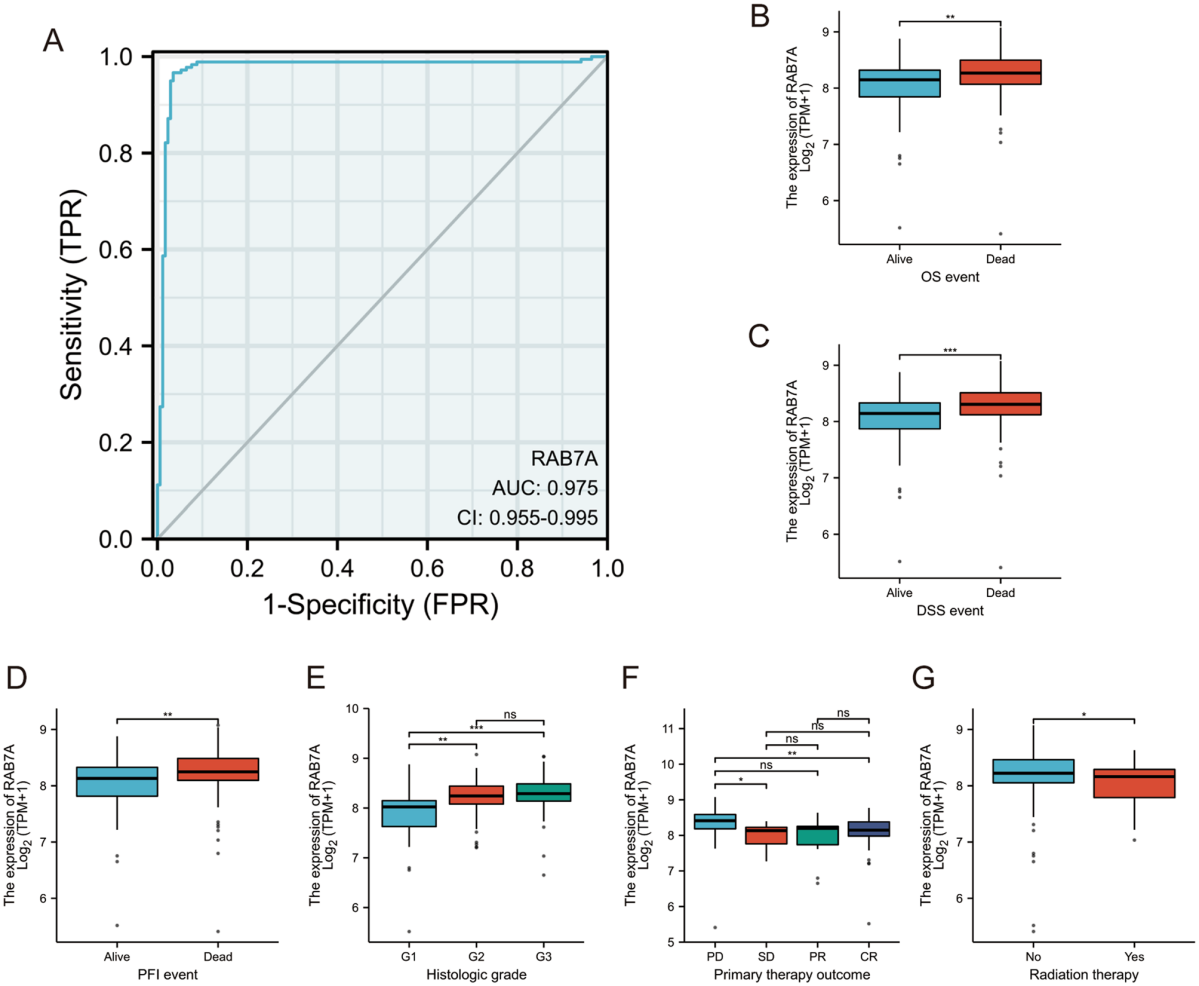
The relationship between RAB7A expression and clinical characteristics. (**A**) Diagnostic effect of RAB7A on Pancreatic adenocarcinoma by ROC analysis. (**B–G**) Relationship between RAB7A expression and OS event, DSS event, PFI event, histologic grade, primary therapy outcome, radiation therapy by Wilcoxon rank sum test.

According to ROC curve analysis, RAB7A shows a high potential for use in diagnosing PAAD patients compared to healthy individuals. The AUC for RAB7A was 0.97, indicating that RAB7A has strong potential for use as a biomarker (Fig. 7A). We compared the expression of RAB7A in individuals with different clinical characteristics using the Wilcoxon rank sum test. The results showed that there was a significant difference in overall survival (P = 0.004), progression-free interval (P = 0.004), and disease-specific survival (P = 0.001) between the pancreatic cancer patients in the high and low RAB7A expression groups. RAB7A was expressed at significantly higher levels in pancreatic cancer patients who had not received radiation therapy than in those who had received radiation therapy. In pancreatic cancer patients with histological grade G1, RAB7A was expressed at relatively low levels. In addition, RAB7A was significantly more highly expressed in patients with pancreatic cancer in the progressive disease (PD) group than in those in the stable disease (SD) and complete response (CR) groups and not significantly different from those in the partial response (PR) group in terms of the outcome of initial treatment (Fig. 7B–G).

### High RAB7A expression affects the prognosis of PAAD patients in different clinical states

**Figure 8:**
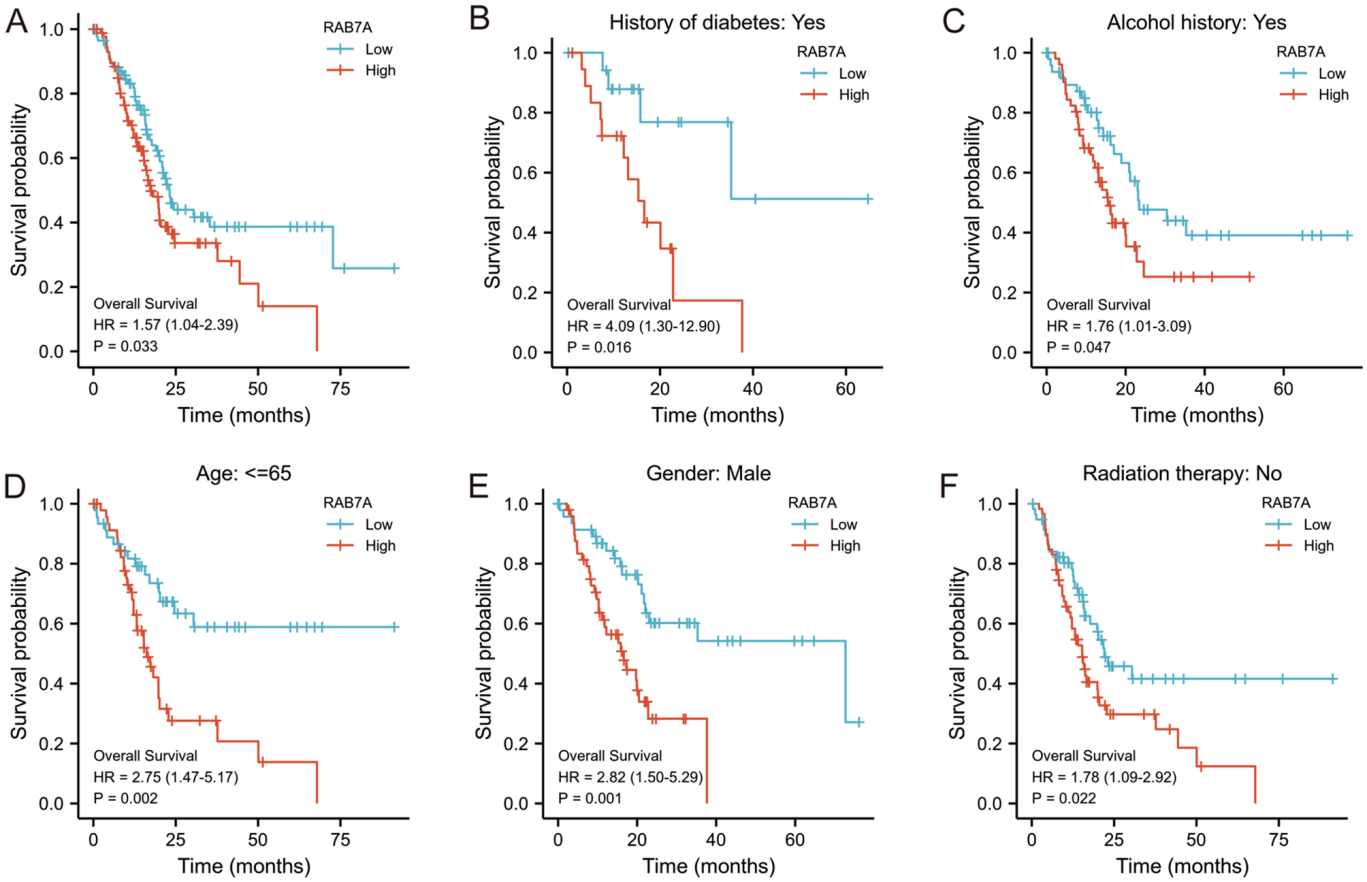
RAB7A overexpression was associated with a poorer OS in PAAD patients. (**A**) Kaplan–Meier curves for all PAAD patients. (**B**) Kaplan–Meier curves for PAAD patients with a history of previous diabetes mellitus. (**C**) Kaplan–Meier curves for PAAD patients with a history of previous alcohol consumption. (**D**) Kaplan–Meier curves for PAAD patients ≤ for 65 years. (**E**) Kaplan–Meier curves for male PAAD patients. (**F**) Kaplan–Meier curves for PAAD patients who did not receive radiotherapy.

Kaplan‒Meier analysis was used to determine the connection between RAB7A expression and the prognosis of patients with PAAD. Patients with high RAB7A expression had a significantly worse prognosis than those with low RAB7A expression in terms of overall survival (hazard ratio [HR], 1.57 (1.04–2.39); P = 0.033) (Fig. 8A). Kaplan‒Meier analysis showed that high expression of RAB7A was associated with poor prognosis in the following subgroups: history of diabetes mellitus (hazard ratio [HR], 4.09 (1.30–12.90); P = 0.016); history of alcohol consumption (hazard ratio [HR], 1.76 (1.01–3.09); P = 0. 047); age up to 65 years (hazard ratio [HR], 2.75 (1.47–5.17); P = 0.002); male patients (hazard ratio [HR], 2.82 (1.50–5.29); P = 0.001); and no previous radiotherapy (hazard ratio [HR], 1.78 (1.09–2.92); P = 0.022) (Fig. 8B–F).

**Figure 9:**
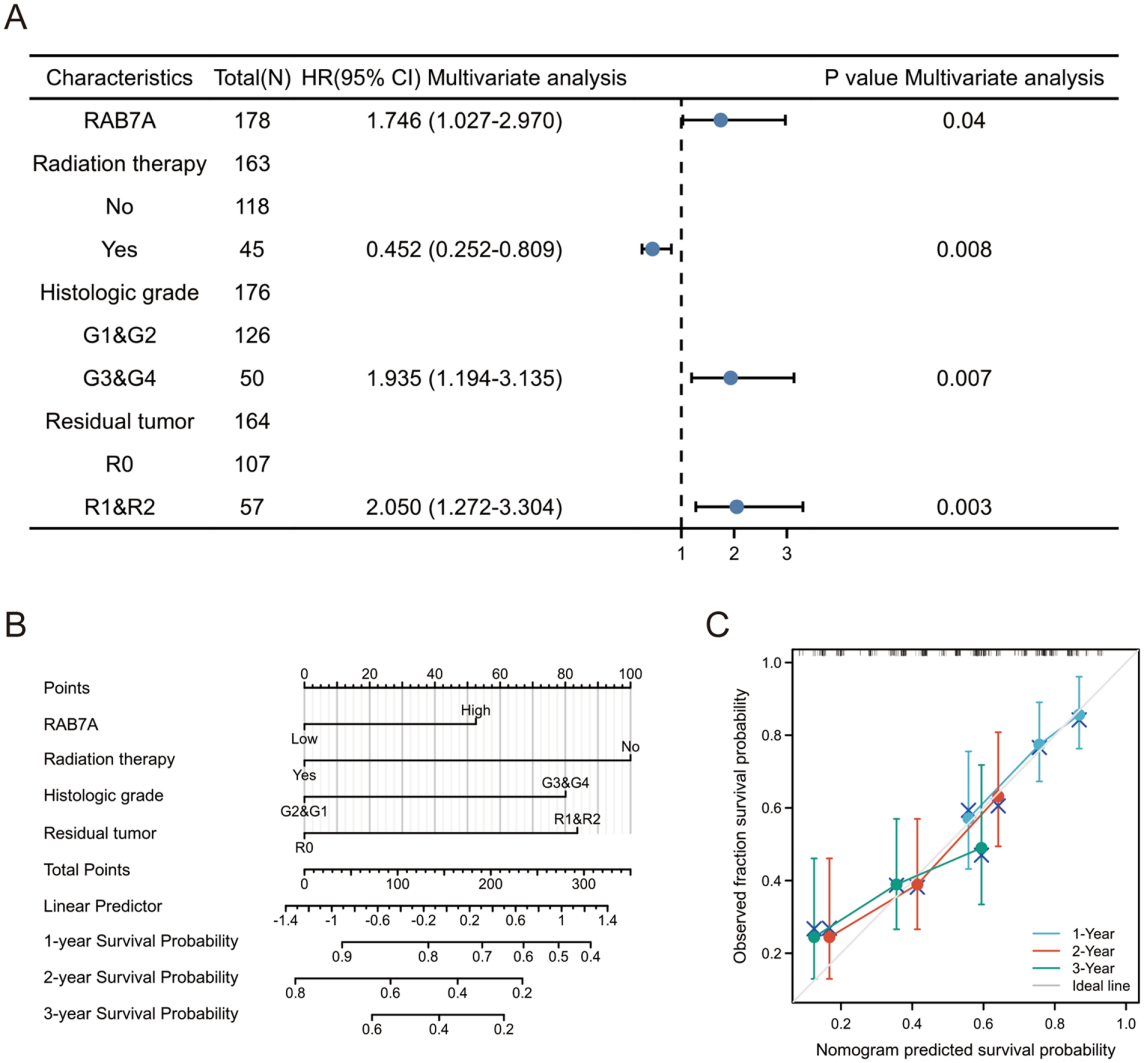
Prognostic prediction model for RAB7A in pancreatic adenocarcinoma. (**A**) The forest plot shows that the expression of RAB7A, the presence or absence of radiotherapy, the early or late histological grading, and the presence or absence of postoperative tumor residue have a significant impact on the patient prognosis. (**B**) Nomogram for predicting 1-, 2- and 3-year OS probabilities in PAAD. (**C**) Calibration plots of the nomogram used to predict 1-, 2- and 3-year OS probabilities.

Thereafter, factors impacting OS were assessed using univariate Cox proportional hazards regression, and RAB7A (high vs. low, P = 0.008) was found to be a predictor of worse OS, as were radiation therapy (yes vs. no, P = 0.013), histologic grade (G1&G2 vs. G3&G4, P = 0.052) and residual tumour (R0 vs. R1&R2, P = 0.028) (Table 1). After that, RAB7A, radiation therapy, histological grade, and residual tumour were all included in multivariate Cox regression, which suggested that they were all independent predictors of poorer OS (P < 0.05) (Fig. 9A).

**Table 1:** Cox regression analysis of factors linked with OS in PAAD, both univariate and multivariate.

Characteristics	Total (N)	Univariate analysis		Multivariate analysis	
		Hazard ratio (95% CI)	P value	Hazard ratio (95% CI)	P value
RAB7A	178	2.089 (1.209–3.611)	**0.008**	1.746 (1.027–2.970)	**0.040**
**Radiation therapy**	163				
No	118	Reference			
Yes	45	0.508 (0.298–0.866)	**0.013**	0.452 (0.252–0.809)	**0.008**
**Histologic grade**	176				
G1&G2	126	Reference			
G3&G4	50	1.538 (0.996–2.376)	**0.052**	1.935 (1.194–3.135)	**0.007**
**Residual tumor**	164				
R0	107	Reference			
R1&R2	57	1.645 (1.056–2.561)	**0.028**	2.050 (1.272–3.304)	**0.003**
**Pathologic stage**	175				
Stage I&Stage II	167	Reference			
Stage III&Stage IV	8	0.673 (0.212–2.135)	0.501		
**Gender**	178				
Female	80	Reference			
Male	98	0.809 (0.537–1.219)	0.311		
**Smoker**	144				
No	65	Reference			
Yes	79	1.086 (0.687–1.719)	0.724		
**Alcohol history**	166				
No	65	Reference			
Yes	101	1.147 (0.738–1.783)	0.542		
**Age**	178				
< = 65	93	Reference			
> 65	85	1.290 (0.854–1.948)	0.227		
**History of diabetes**	146				
No	108	Reference			
Yes	38	0.927 (0.532–1.615)	0.790		
**History of chronic pancreatitis**	141				
No	128	Reference			
Yes	13	1.177 (0.562–2.464)	0.666		
**Family history of cancer**	110				
No	47	Reference			
Yes	63	1.117 (0.650–1.920)	0.689		

Significant values are in bold.

### Prognostic model of RAB7A in PAAD

Based on the results of Cox regression analysis, we constructed a nomogram using the rms R package to better predict the prognosis of PAAD patients (Fig. 9B). Four independent prognostic factor variables, namely, RAB7A expression, radiation therapy, histological grade and residual tumour, were included in the model. These variables were allocated points using a point scale derived from multivariate Cox analysis. To identify the points for the variables, straight lines were plotted upwards, and the sum of the points allocated to each variable was modified to a range of 0–100. The final score can be calculated by adding the points for each variable. A line can be drawn straight down from the total point axis to assess the probability of survival for PAAD patients at 1, 2 and 3 years. A vertical line drawn along the 200 towards the endpoint axis on the total point axis indicates 1-year survival < 70% and both 3-year and 5-year survival < 40%. The calibration curves showed that the predicted values of the OS nomogram for PAAD patients were largely consistent with the observed values (Fig. 9C).

## Discussion

RAB7A is a small molecule GTPase of the RAB family that is widely expressed^23^. Considering that RAB7A is involved in many cellular processes, it is not unreasonable to speculate that its expression, with altered biochemical properties, could lead to tumorigenesis. Several studies have shown increased expression of RAB7A in cholangiocarcinoma cells following the induction of epithelial–mesenchymal transition (EMT) and invasion by tumour necrosis factor-α (TNF-α) stimulation^24^. Studies of several tumours, such as thyroid adenomas and ovarian/primary peritoneal plasmacytoma, have assessed the expression and function of RAB7A in tumour development^25,26^. However, nothing is known about the expression of RAB7A in PAAD, let alone its prognostic significance.

The most important finding of this study was that high expression of RAB7A in PAAD was associated with poor patient prognosis. By GSEA, low RAB7A expression was associated with the PPAR alpha pathway and PPAR signalling pathway. PPAR agonists are commonly used in the treatment of diabetes mellitus. As the target organ of both diabetes and PAAD is the pancreas, some studies have reported a therapeutic effect of PPAR agonists on PAAD^27^. Meanwhile, PPARs are involved in cell differentiation, development, metabolism and tumorigenesis. PPAR-γ has been found to be a risk factor for the development of cholangiocarcinoma, gastric cancer and breast cancer^27^. Inhibitors of PPAR-γ are beneficial for the treatment of advanced PAAD^28^. In view of the above reports, the relationship between PPAR pathway genes and their genetic variants and pancreatic cancer is not very clear, and further exploratory studies are needed. Conversely, high expression of RAB7A was associated with the regulation of TP53 activity, the pancreatic adenocarcinoma pathway, pancreatic cancer, the regulation of RAS by gaps, and oncogenic MAPK signalling. The RAS/mitogen-activated protein kinase (MAPK) pathway plays a central role in human cancers. It is highly activated in a variety of tumours, and many of its components have been identified as oncogenes^29^. KRAS mutations occur early in the development of PAAD and are found in 90% of PAAD patients. As PAAD continues to progress, more genes, such as CDKN2A and TP53, are mutated. This shows that RAB7A, by altering tumorigenesis-related pathways in PAAD, is not only a possible prognostic biomarker but also a promising therapeutic target. Additionally, here, RAB7A was discovered to be connected with these pathways and may play a role in the beginning and maintenance of PAAD; however, more research is needed to confirm our findings and to investigate the particular regulatory mechanisms of RAB7A and these pathways. GSEA also further confirmed the plausibility of the PPI results, e.g. ABL protein enrichment in the pancreatic adenocarcinoma pathway, pancreatic cancer, CPB1 enrichment in the metabolism of angiotensinogen to angiotensins, and peptide hormone metabolism.

High RAB7A expression was correlated with high Th2 cell expression in immune cell infiltration analysis. The involvement of Th2 cells in the development of allergic diseases and in immunity to parasites has been widely demonstrated. Several studies have reported the impact of Th2 cell-mediated type 2 immunity on the tumour microenvironment (TME) and tumour progression. Th2 cells and type 2 immunity have been demonstrated to play a role in tumour immune surveillance, for example, by lowering the growth of existing tumors^30,31^. More importantly, tumour clearance was reduced in mice lacking the Th2 cytokines IL-4 and IL-5. Injection of IL-4 enhanced tumour clearance and resulted in an increase in the infiltration of eosinophils, macrophages, neutrophils, and some lymphocytes. Additionally, monoclonal antibodies neutralizing IL-5 restored tumour development^32–34^. These studies suggest that Th2 cytokines are important in antitumour immunity.

Notably, it has also been shown that Th2 immunity is associated with cancer development, progression and metastasis. For example, one study showed that in mice implanted with Th2 cells, lung cancer slowly remitted as the transition from Th2 cells to Th1 cells occurred^35^. Th2 cells in the TME have been linked to the progression of breast cancer and cervical cancer, according to research^36^. Additionally, it has been demonstrated that type 2 immunity promotes metastases in breast, colorectal, and lung cancers^37–40^. In conclusion, numerous studies have shown that Th2 cells have both tumour-promoting and tumour-suppressing roles. The role of Th2 cells and type 2 immunity in promoting or suppressing tumour growth appears to be highly dependent on the nature of the tumour and the stage of the tumour. In the present study, infiltration of Th2 cells was positively correlated with RAB7A expression. High RAB7A expression was associated with poor prognosis in PAAD patients according to Kaplan‒Meier survival analysis. On the other hand, the link between Th2 cells and PAAD has not been extensively explained. Based on our findings and those of the studies mentioned above, the interaction between RAB7A and Th2 cells, as well as whether RAB7A and Th2 cells are involved in immunological escape in PAAD, still needs to be investigated further.

The most clinically significant conclusion was that higher RAB7A expression was linked to lower patient survival. In addition, according to the Cox analysis in this study, RAB7A may be an independent predictor of poor prognosis in PAAD following adjustment for some clinical features. Multifactorial Cox regression analysis showed that high RAB7A expression was an independent prognostic factor for worse OS, in addition to radiotherapy, histological grading and postoperative residual tumour. A more accurate prognostic prediction model was obtained by combining RAB7A with radiotherapy, histological grading and postoperative residual tumour to construct a prognostic nomogram, which had a C-index of 0.665 (0.631–0.699). In the calibration plots, good agreement between the predictions of the present model and the actual observations of 1-year OS were observed. Thus, our model is capable of producing personalized scores for PAAD patients. The bias between the actual observation of 2-year and 3-year OS probabilities with increasing time may be related to the overly complex factors affecting the prognosis of pancreatic cancer patients. However, the relatively small sample size was a shortcoming of the present study. Therefore, subsequent studies should expand the sample size to ensure the reliability and representativeness of the hypotheses and results of this study. In addition, a series of experiments should be performed to investigate the regulatory mechanisms between RAB7A and the pancreatic cancer-related (e.g. MAPK, KRAS, PPAR) pathways screened by GSEA. A significant amount of work has recently been planned in the laboratory.

## Conclusion

In this study, we combined public databases and performed an in-depth analysis of PAAD and adjacent tissues separately. This study is the first to show that PAAD patients had higher RAB7A expression, which is linked to a poor prognosis. The apparent upregulation of multiple classic cancer pathways, such as RAS, MAPK and PPAR, indicates that these pathways may be important in the regulation of RAB7A in PAAD. Our prognostic model based on high RAB7A expression has good stability and validity. Our findings provide comprehensive evidence that high RAB7A expression is a poor prognostic factor in pancreatic cancer. These clues set the stage for a further understanding of the integrative role of RAB7A in cells and RAB7A as a therapeutic target.

## Supplementary Information

Supplementary Information 1.

Supplementary Information 2.

Supplementary Information 3.

Supplementary Information 4.

Supplementary Figure 1.

## Data Availability

RNAseq data in TPM format for TCGA and GTEx for pan-cancer and PAAD were provided by UCSC XENA (https://xenabrowser.net/datapages/), which are publicly available.

## Abbreviations

RAB7A: RAS-related protein Rab-7aRAB
PAAD: Pancreatic adenocarcinoma
GTPases: Guanosine triphosphatases
CT: Cycle threshold
WB: Western blot
HPA: Human protein atlas
TCGA: The cancer genome atlas
GTEx: Genotype-tissue expression
DEGs: Differentially expressed genes
GSEA: Gene set enrichment analysis
ssGSEA: Single-sample gene set enrichment analysis
GO: Gene ontology
CC: Cellular component
MF: Molecular function
BP: Biological process
KEGG: Kyoto encyclopedia of genes and genomes
STRING: Search tool for the retrieval of interacting genes
PPI: Protein–protein interaction
Tgd: T gamma delta
Tfh: T follicular helper
Tem: T effector memory
Tcm: T central memory
aDC: Activated DC
iDC: Immature DC
pDC: Plasmacytoid DC
ROC: Receiver operating characteristic
PD: Progressive disease
SD: Stable disease
CR: Complete response
PR: Partial response
OS: Overall survival
PFI: Progression-free interval
DSS: Disease specific survival
AUC: Area under the curve
EMT: Epithelial-mesenchymal transition
TNF-α: Tumor necrosis factor- α
MAPK: Mitogen-activated protein kinase
TME: Tumor microenvironment
