# Supplementary figures and images for: High RAS-related protein Rab-7a (RAB7A) expression is a poor prognostic factor in pancreatic adenocarcinoma
Source: Sci Rep. 2022 Oct 19;12:17492. doi: 10.1038/s41598-022-22355-1 (PMC9582019; doi:10.1038/s41598-022-22355-1)

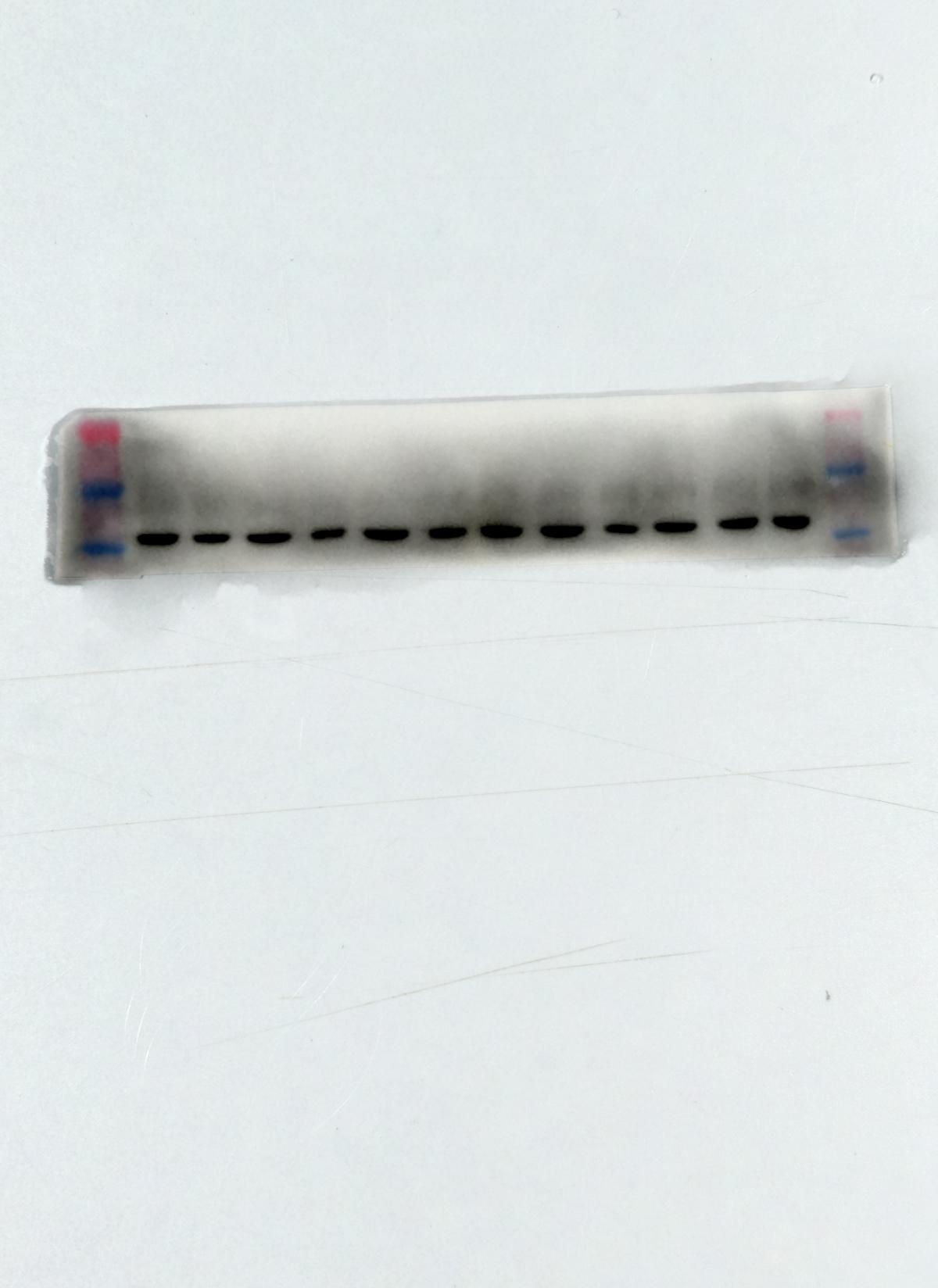

Supplement: Supplementary file 1 — Supplementary Information 1. [file 41598_2022_22355_MOESM1_ESM.jpg]

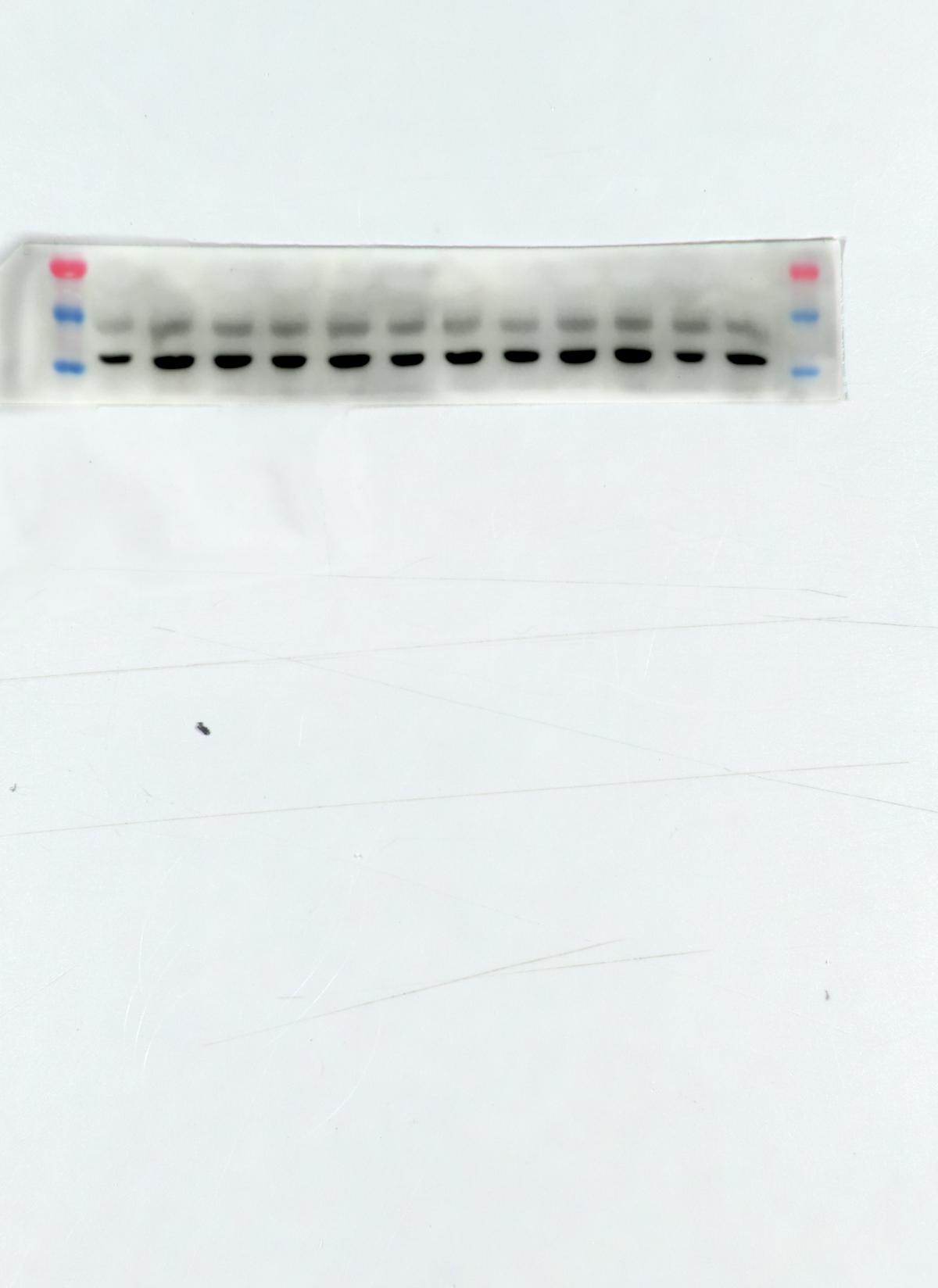

Supplement: Supplementary file 2 — Supplementary Information 2. [file 41598_2022_22355_MOESM2_ESM.jpg]

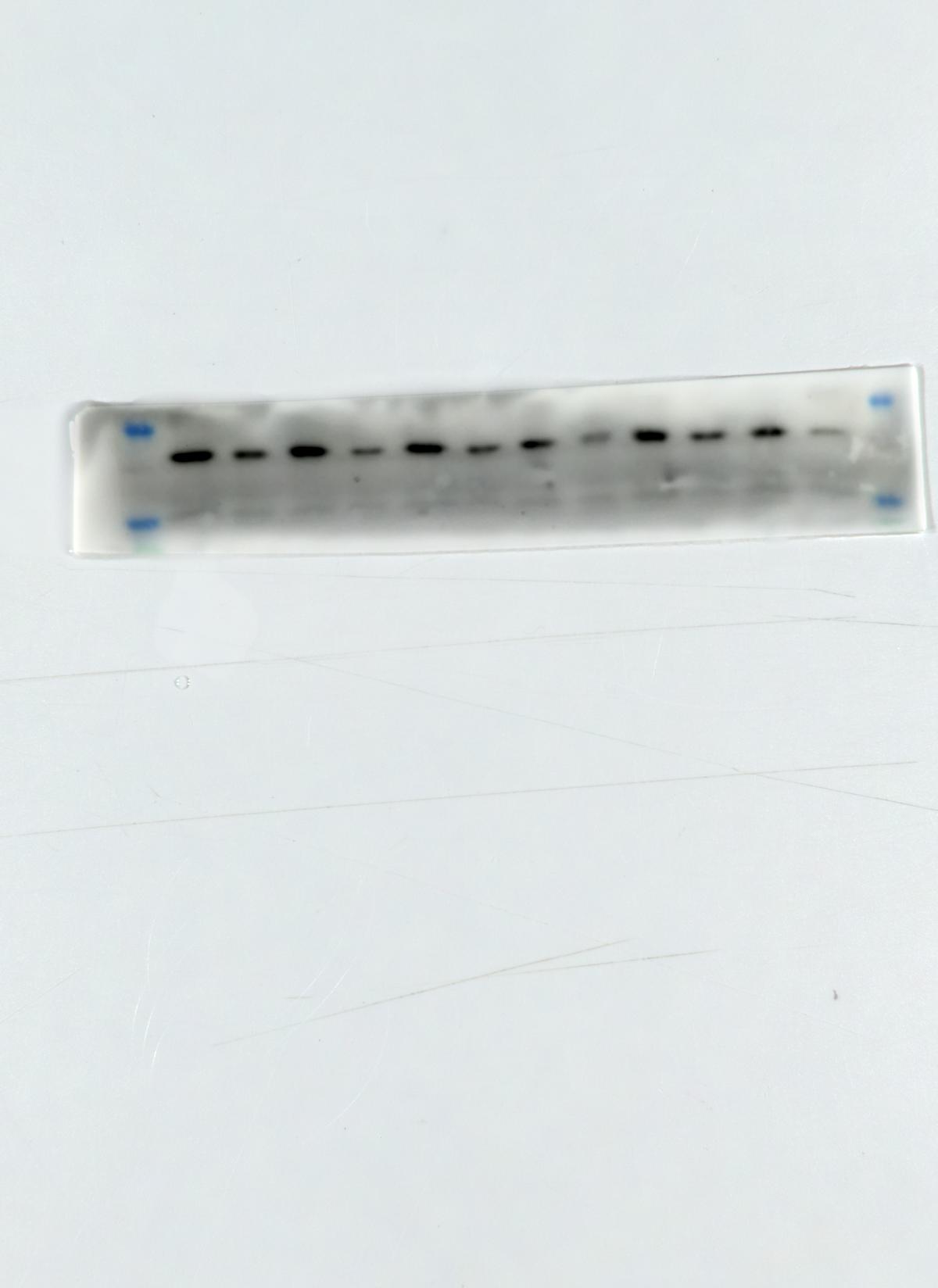

Supplement: Supplementary file 3 — Supplementary Information 3. [file 41598_2022_22355_MOESM3_ESM.jpg]

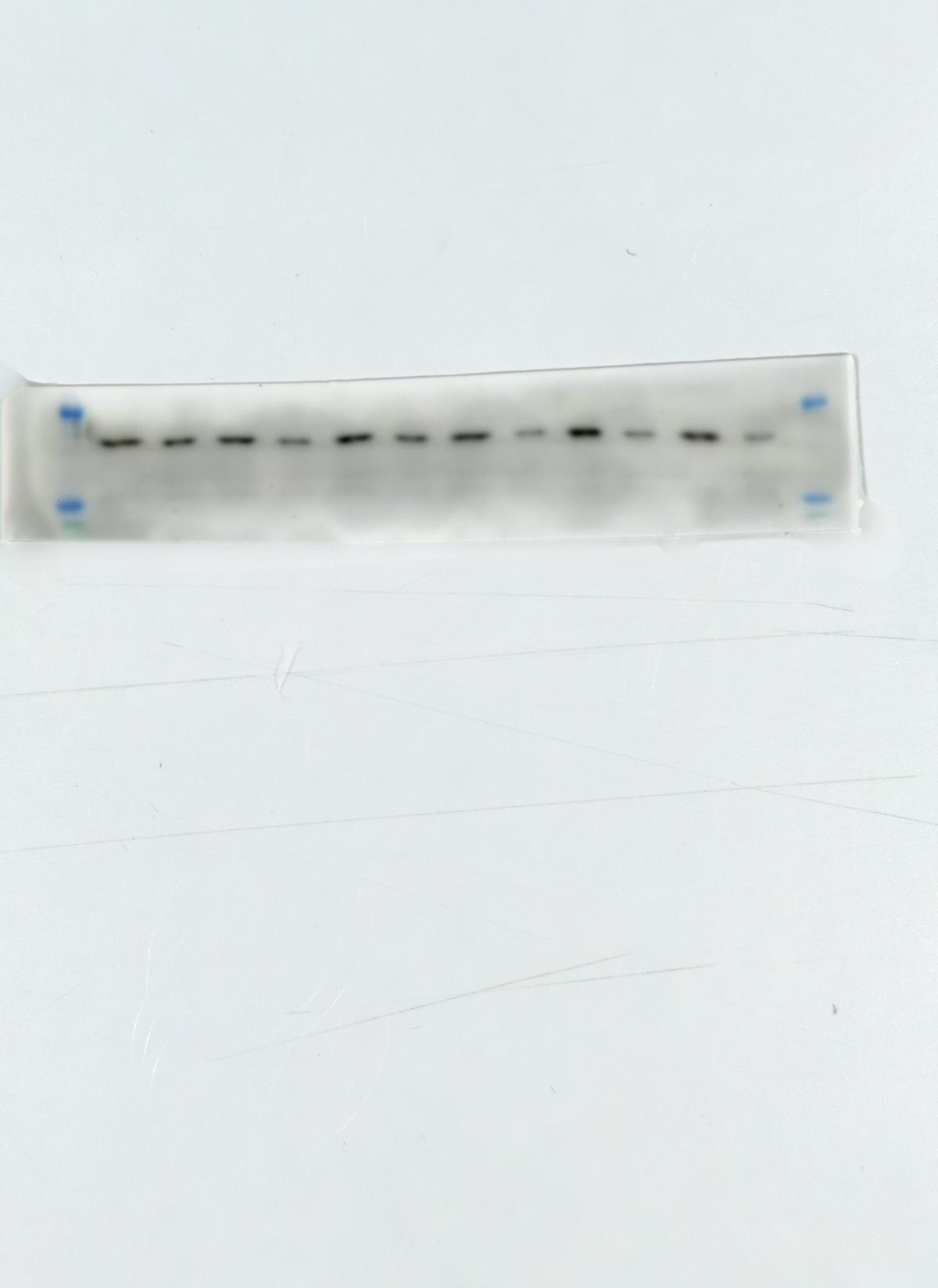

Supplement: Supplementary file 4 — Supplementary Information 4. [file 41598_2022_22355_MOESM4_ESM.jpg]
