# Supplementary material for: High RAS-related protein Rab-7a (RAB7A) expression is a poor prognostic factor in pancreatic adenocarcinoma
Source: Sci Rep. 2022 Oct 19;12:17492. doi: 10.1038/s41598-022-22355-1 (PMC9582019; doi:10.1038/s41598-022-22355-1)
Supplement: Supplementary file 5 — Supplementary Figure 1. [file 41598_2022_22355_MOESM5_ESM.pdf]

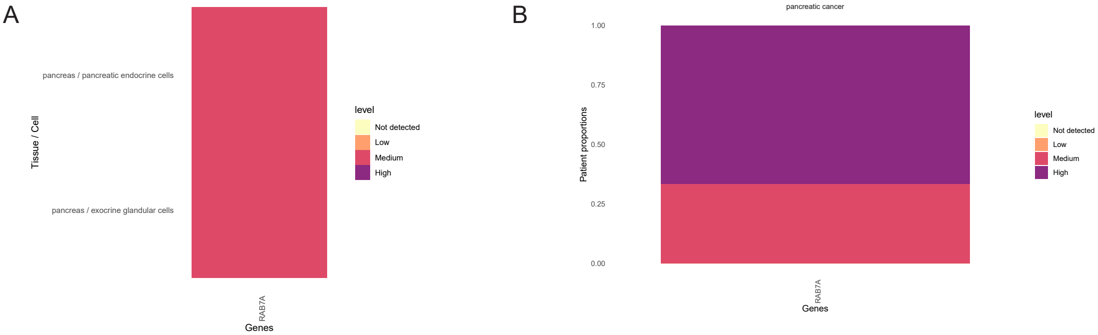

Supplementary Fig. 1 The expression of RAB7A in all normal pancreatic tissues versus all pancreatic cancer tissues on HPA. **A** RAB7A was expressed at moderate intensity in all normal pancreatic tissues. **B** The majority of RAB7A is highly expressed in pancreatic cancer tissues.
